# Demography, common disorders and mortality of Shih Tzu dogs under primary veterinary care in the UK

**DOI:** 10.1186/s40575-023-00135-y

**Published:** 2024-01-24

**Authors:** Fiona Dale, Dave C. Brodbelt, Gabriella West, David B. Church, Yan Hui Lee, Dan G. O’Neill

**Affiliations:** 1 Pathobiology and Population Sciences, The Royal Veterinary College, North Mymms, Hawkshead Lane, Hatfield, AL9 7TA Herts UK; 2 Clinical Science and Services, The Royal Veterinary College, North Mymms, Hawkshead Lane, Hatfield, AL9 7TA Herts UK

**Keywords:** VetCompass, Electronic patient record, EPR, Breed, Dog, Epidemiology, Primary-care, Veterinary, Pedigree, Purebred, Shih Tzu

## Abstract

**Background:**

Shih Tzus are a popular dog breed in the UK although there is relatively little reported information on their health. This study aimed to characterise the demography, common disorders and mortality of Shih Tzus under primary veterinary care during 2016 in the UK using de-identified clinical records from the VetCompass™ Programme.

**Results:**

The study population of 336,865 dogs under veterinary care during 2016 included 11,082 Shih Tzus (3.3%). The median age was 4.1 years (IQR: 2.1–7.1, range: 0.3–20.4) and mean adult bodyweight was 7.9 kg (SD: 1.9 kg). Annual proportional births increased from 2.2% of all dog births in 2005 to 3.8% in 2013, dropping to 3.3% by 2016. From a random subset of 2,423 Shih Tzus that had information extracted on disorders diagnosed during 2016, the most prevalent fine-level precision disorders were periodontal disease (*n* = 229, prevalence 9.5%, 95% CI: 8.4–10.7), anal sac impaction (180, 7.4%, 95% CI: 6.5–8.5) and ear disorders (134, 5.5%, 95% CI: 4.7–6.5). The most prevalent grouped-level precision disorders were cutaneous (*n* = 402, prevalence: 16.6%, 95% CI: 15.2–18.1), dental (322, 13.3%, 95% CI: 12.0–14.7), and ophthalmological (289, 11.9%, 95% CI: 10.7–13.3). Males were more likely than females to be diagnosed with skin disorders (*P* = 0.007) and musculoskeletal disorders (*P* = 0.010) while females were more likely than males to be diagnosed with hernias (*P* = 0.005). The median age of death was 12.7 years (IQR 8.7–14.3, range 2.0–19.9) and did not differ statistically between males and females. The most common grouped causes of death were enteropathy (7.9%, 95% CI: 3.9–15.4), heart disease (7.9%, 95% CI: 3.9–15.4) and poor quality of life (7.9%, 95% CI: 3.9–15.4).

**Conclusions:**

Periodontal disease, anal sac impaction and ear disorders were identified as common health issues. Shih Tzus had higher prevalence of anal sac impaction, umbilical hernias and eye problems than reported previously in dogs overall, suggesting potential predispositions. Shih Tzus appear to be relatively long-lived compared to previous reports of lifespan in dogs overall. The results can inform veterinarians and owners on priority disorders for monitoring to protect welfare. Oral hygiene was highlighted as a healthcare priority.

## Abstract (plain language summary)

The Shih Tzu is a popular small breed of dog in the UK. However, there is little information on common health conditions affecting this breed. This study aimed to describe the demography, common disorders, and causes of death of Shih Tzus in the UK in 2016 using the VetCompass™ Programme, which holds a database of anonymized clinical records of dogs attending primary-care veterinary practices in the UK.

The study included 11,082 Shih Tzus (3.3%) from 336,865 dogs under veterinary care in the UK in VetCompass™ in 2016. The popularity of the Shih Tzu increased slightly from 2005 to 2013 but showed signs of decline towards 2016. The average age of Shih Tzus in the study was 4.1 years and the average adult bodyweight was 7.9 kg. The most common disorders affecting Shih Tzus in the study were periodontal disease (inflammation of the gums and tissue around the teeth, affecting 9.5% of Shih Tzus), anal sac impaction (7.4%) and ear disorders (5.5%). Male Shih Tzus were more likely than females to have skin and musculoskeletal disorders (conditions affecting bones, muscles and joints), while female Shih Tzus were more likely be diagnosed with hernias (when internal tissues push out through weakened muscle tissues). Shih Tzus had higher prevalence of anal sac impaction, umbilical hernias and eye problems than previously reported in dogs overall, suggesting potential predispositions. The average age at death of Shih Tzus was 12.7 years of age and the most common causes of death were enteropathy (gastrointestinal diseases causing signs such as diarrhoea and vomiting, 7.9%), heart disease (7.9%) and poor quality of life (7.9%).

This study demonstrated the value of analysis of veterinary clinical records to increase our understanding of common health problems affecting dogs in the UK. The results can help both current and prospective Shih Tzu owners, and veterinarians, in prioritising preventative healthcare for Shih Tzus. For example, the most common disorder in Shih Tzus was periodontal disease, suggesting that increased preventative dental checkups and toothbrushing could improve welfare, especially if implemented from an early age.

## Background

The Shih Tzu, previously known as the Lhasa Lion Dog, is a small breed of dog believed to have originated in China [1]. The United Kingdom (UK) Kennel Club describes the pedigree subset of the breed as ‘sturdy’, ‘friendly’ and ‘intelligent’, with a bodyweight of 4.5 to 8.0 kg. In the UK, the Shih Tzu was the 13^th^ most popular dog breed in 2021 based on an online survey of dog owners [2]. However, since 2012, the number of Shih Tzu registrations with the UK Kennel Club have been decreasing, suggesting dwindling popularity of the registered subset of the breed over the past decade [3]. Indeed, UK Kennel Club registrations of Shih Tzus more than halved from 2013 to 2022 (4,319 to 1,906 registrations) [3, 4]. Previous analysis of mortality data from UK primary-care veterinary records reported a median age at death of 13.3 years for Shih Tzus [5]. A more recent study into longevity of dogs in the UK reported a life expectancy from age 0 for Shih Tzus of 11.05 years that was comparable to that reported for UK dogs overall (11.23 years) and for other small breeds (e.g. Yorkshire Terrier (12.54 years), Cavalier King Charles Spaniel (10.45 years)) [6] suggesting that Shih Tzu were not atypical in terms of longevity [6].

Literature on disorder prevalence and predispositions in Shih Tzus is relatively limited. A cross-sectional survey of owners of pedigree dogs registered with the UK Kennel Club reported that 39.60% of Shih Tzus had ≥ 1 reported disease/condition [7]. In that study, compared to dogs overall, Shih Tzus had significantly higher prevalence for anal sac impaction, corneal ulceration, intervertebral disc disorder, umbilical hernia, and unspecified eye disorders [7]. Increased prevalence in Shih Tzus of intervertebral disc disease had previously been reported in a retrospective study of cases in the United States (US) and Canada [8]. Shih Tzus were among the most commonly affected breeds diagnosed with adult-onset demodicosis, in a retrospective study of 431 dogs presented to a US teaching hospital over a seven-year period [9]. Previous analysis of UK primary-care data reported that Shih Tzu dogs younger than two years were less likely than crossbreed dogs to develop demodicosis, but had increased odds among dogs older than four years of age [10]. Shih Tzus have also been reported at increased risk of anal sac disorders compared to crossbreed dogs in cohort analyses of dogs under primary veterinary care during 2013 [11].

The UK Kennel Club classes the Shih Tzu as a Breed Watch category 1 breed, defined as currently having no known health concerns which require special attention from judges [12]. There are no health screening or DNA testing specifically recommended by The Kennel Club for Shih Tzus, but prospective breeders and owners are directed to information on health problems that affect flat-faced dogs more generally [1, 12]. Based on their short muzzles, Shih Tzus are considered a brachycephalic breed [13]. Because of their shortened muzzles, brachycephalic breeds (flat-faced dogs) often have excessive facial skin-folds making them likely to experience problems such as skin fold dermatitis [14], while their extreme skull conformation can also promote dental issues including malocclusion [15]. In addition, shallow orbits mean that the eyes of dogs with brachycephaly protrude more from their sockets than non-brachycephalic dogs making these dogs susceptible to ophthalmic disorders such as corneal ulceration, globe prolapse [15, 16], keratoconjunctivitis sicca [17], and prolapse of the nictitating membrane gland [18]. A recent study of primary veterinary practice records reported that Shih Tzus were predisposed to prolapse of the nictitating membrane gland with an annual breed prevalence of 0.19% [18]. Furthermore, a cross sectional study based on referral veterinary clinical data reported that brachycephalic dogs were 20 times more likely to be affected by corneal ulcers than non-brachycephalic dogs [19]. Previous research has identified that Shih Tzus were amongst breeds with the highest prevalence of corneal ulcerative disease, alongside other brachycephalic breeds such as Pugs and English Bulldogs and that brachycephalic breeds had 11.18 times the odds of corneal ulcerative disease compared to crossbreed dogs [20]. Similarly, Shih Tzus represented the majority of ulcerative keratitis cases in a study of 32 cases [21]. The extreme body conformation of brachycephalic dogs, specifically short muzzles, can also promote brachycephalic obstructive airway syndrome (BOAS) where the upper respiratory airway is either partially or completely obstructed resulting in difficulties breathing [13]. BOAS has been reported in Shih Tzus [22], as well as several over brachycephalic breeds including the Pug, Boston Terrier, French Bulldog [22]. Clarifying disorder prevalence in Shih Tzus, a brachycephalic breed, could assist veterinarians and breeders in prioritising targeted healthcare.

The reporting of sex and age-related differences within breeds can help veterinarians focus on preventative healthcare unique to specific target groups as appropriate [23, 24]. Previous work has reported differences in lifespan and disorder prevalence between the sexes and age groups of dog breeds, for example Chihuahuas and Miniature Schnauzers [24, 25]. For Shih Tzus specifically, there has been relatively little research into age or sex-specific differences; however a recent study did report differences in the ocular surface between male and female Shih Tzus presenting to ophthalmic departments in the US, with male Shih Tzus having a greater palpebral fissure length than females [26].

The electronic patient records (EPRs) of dogs under primary-care hold routine information on demographics, e.g. breed, sex-neuter status, as well as disorder occurrence and mortality [27]. EPRs can be utilised as a secondary data source in veterinary research that offers generalisability of results to the general UK dog population, given that 77% of the UK dog population are estimated to be registered in primary veterinary care [28]. Using veterinary clinical data from the VetCompass™ Programme [29], this study aimed to describe the demography, common disorders and mortality of the general population of Shih Tzus under veterinary care in the UK in 2016. The results from the current study could provide a reliable framework to assist reforms in breeding practices and ultimately contribute to improved health and welfare of Shih Tzus.

## Materials and methods

The study population included all dogs under primary veterinary care at clinics participating in the VetCompass™ Programme during 2016. Dogs under veterinary care were defined as those with either a) at least one EPR (free-text clinical note, treatment or bodyweight) recorded during 2016 or b) at least one EPR recorded both before and after 2016. The VetCompass™ Programme collates de-identified EPR data from primary-care veterinary practices in the UK for epidemiological research [29]. Data fields available for VetCompass™ researchers include a unique animal identifier along with species, breed, date of birth, colour, sex, neuter status and bodyweight, and clinical information from free-form text clinical notes, summary diagnosis terms (VeNom codes [30]) and treatments with relevant dates.

A cohort study design with a cross-sectional analysis was used to estimate the one-year period prevalence of the most commonly diagnosed disorders [31]. Sample size calculations estimated that 2,369 dogs were needed to report prevalence for a disorder diagnosed in 2.0% of dogs with 0.5% margin of error to a 95% confidence level from a population of 11,082 dogs [32]. Ethics approval was obtained from the RVC Ethics and Welfare Committee (reference number 2015/1369).

Dogs recorded as Shih Tzu breed were categorised as Shih Tzu and all remaining dogs were categorised as non-Shih Tzu. *All-age Bodyweight* (Kg) included all available unique bodyweight and date combinations. *Adult Bodyweight* (Kg) described the mean bodyweight recorded from all bodyweight data for each dog aged over 18 months and was categorised into 7 groups for both males and females (< 5.0, 5.0 to < 6.0, 6.0 to < 7.0, 7.0 to < 8.0, 8.0 to < 9.0, 9.0 to < 10.0, ≥ 10.0). N*euter* described the status of the dog (entire or neutered) at the final EPR. *Age* described the age at the final date under veterinary care during 2016 (December 31^st^, 2016) and was categorised into 6 groups (< 1.0, 1.0 to < 2.0, 2.0 to < 4.0, 4.0 to < 6.0, 6.0 to < 9.0, ≥ 9.0) following visual inspection of the data.

The list of unique Shih Tzu animal identification numbers was randomly ordered and the clinical records of a random subset of animals were reviewed manually in detail. The most definitive diagnoses recorded were extracted, based on all disorders with evidence in the clinical records that the disorder existed during 2016. These disorders were manually linked to the most appropriate VeNom term as previously described [30]. Elective (e.g., neutering) or prophylactic (e.g., vaccination) clinical events were not included. No distinction was made between pre-existing and incident disorder presentations. Disorders described within the clinical notes using presenting sign terms (e.g. ‘vomiting’ or 'vomiting and diarrhoea'), but without a formal clinical diagnostic term recorded, were included using the first sign listed (e.g. vomiting). Mortality data (recorded cause, date and method of death) were extracted on all deaths at any date during the available EPR data. The extracted diagnosis terms were mapped to a dual hierarchy of precision for analysis: fine-level precision and grouped-level precision as previously described [33]. Briefly, fine-level precision terms described the original extracted terms at the maximal diagnostic precision recorded within the clinical notes (e.g. *inflammatory bowel disease* would remain as *inflammatory bowel disease*). Grouped-level precision terms mapped the original diagnosis terms to a general level of diagnostic precision (e.g. *inflammatory bowel disease* would map to *gastro-intestinal*). The epidemiological unit for the current study was each individual Shih Tzu dog for the single year of the study (2016). No information was extracted on the owner’s original rationale (e.g., seeking prophylactic care versus presenting a dog with prior awareness of health issues) for seeking veterinary care across the range of veterinary interactions that may have occurred during this year of interest.

Following data checking for internal validity and cleaning in Excel (Microsoft Office Excel 2013, Microsoft Corp.), analyses were conducted using SPSS Version 28. The sex, neuter status, age and adult bodyweight for Shih Tzus under veterinary care during 2016 were described. Annual proportional birth rates described the relative proportion of Shih Tzus compared with all dogs that were born in each year from 2005–2016 from the overall cohort of dogs under veterinary care in 2016. All-age bodyweight data with their associated dates were used to generate separate male and female bodyweight growth curves in SPSS for Shih Tzus by plotting age-specific bodyweights and were overlaid with a loess line using uniform kernel setting fitting 50% of points. Continuous variables that were normally distributed were described using mean (standard deviation [SD]). Non-normal variables were described with a median (interquartile range [IQR] and range) [34].

One-year (2016) period prevalence values and proportional mortality were reported along with 95% confidence intervals (CI) that described the probability of diagnosis at least once during 2016. The CI estimates were derived from standard errors based on approximation to the normal distribution for disorders with ten or more events [34] or the Wilson approximation method for disorders with fewer than ten events [35]. Prevalence values were reported overall and separately for males and females. The prevalence of a combined list of the 10 most common disorders recorded at a fine-level of diagnostic precision within each of three age bands (under 2 years, 2–7 years, over 7 years) were presented and comparison of prevalence between the three age bands was conducted using chi-squared tests. The chi-square test was used to compare categorical variables and the Students t-test or Mann–Whitney U test to compare continuous variables as appropriate [34]. Statistical significance was set at the 5% level.

## Results

### Demography

**Fig. 1:**
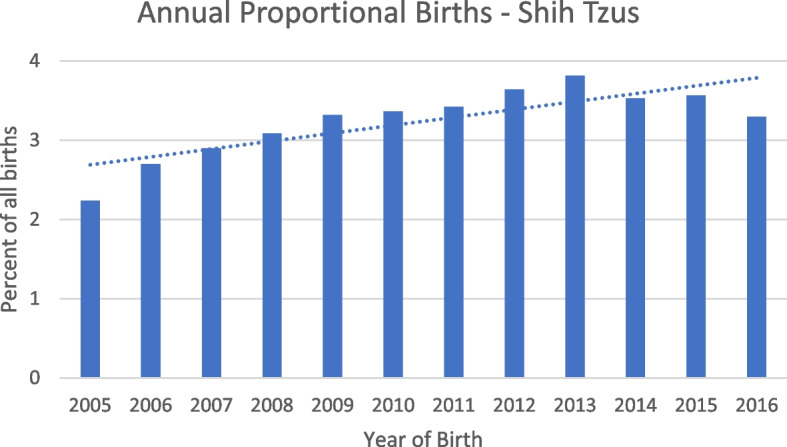
Annual proportional birth rates (2005–2016) for Shih Tzus (*n* = 10,213) among all dogs (*n* = 336,865) attending UK primary-care veterinary clinics participating in the VetCompass™ Programme

**Fig. 2:**
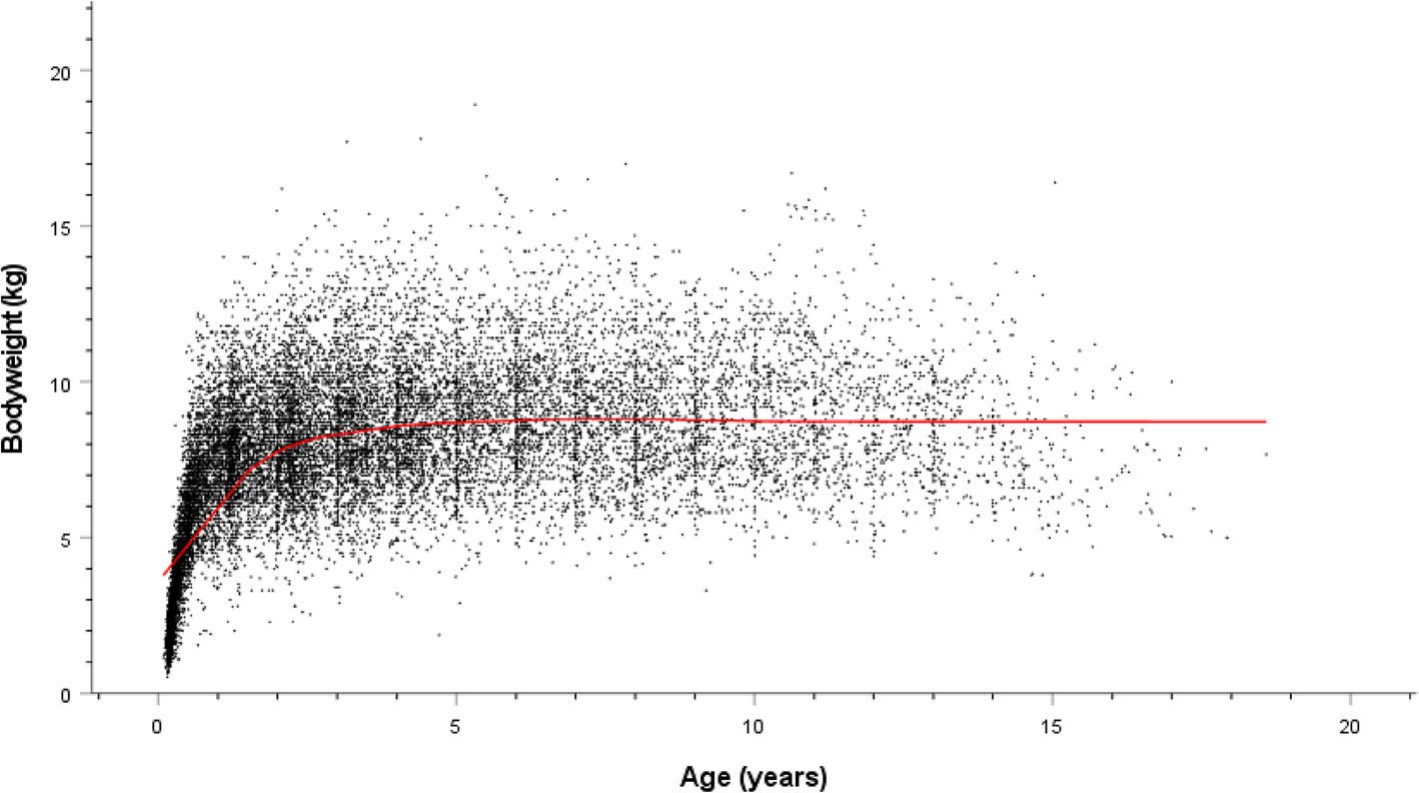
Bodyweight growth curve line plot overlaid with a loess line (uniform kernel, fitting 50% of points) for male Shih Tzus attending UK primary-care veterinary clinics participating in the VetCompass™ Programme

**Fig. 3:**
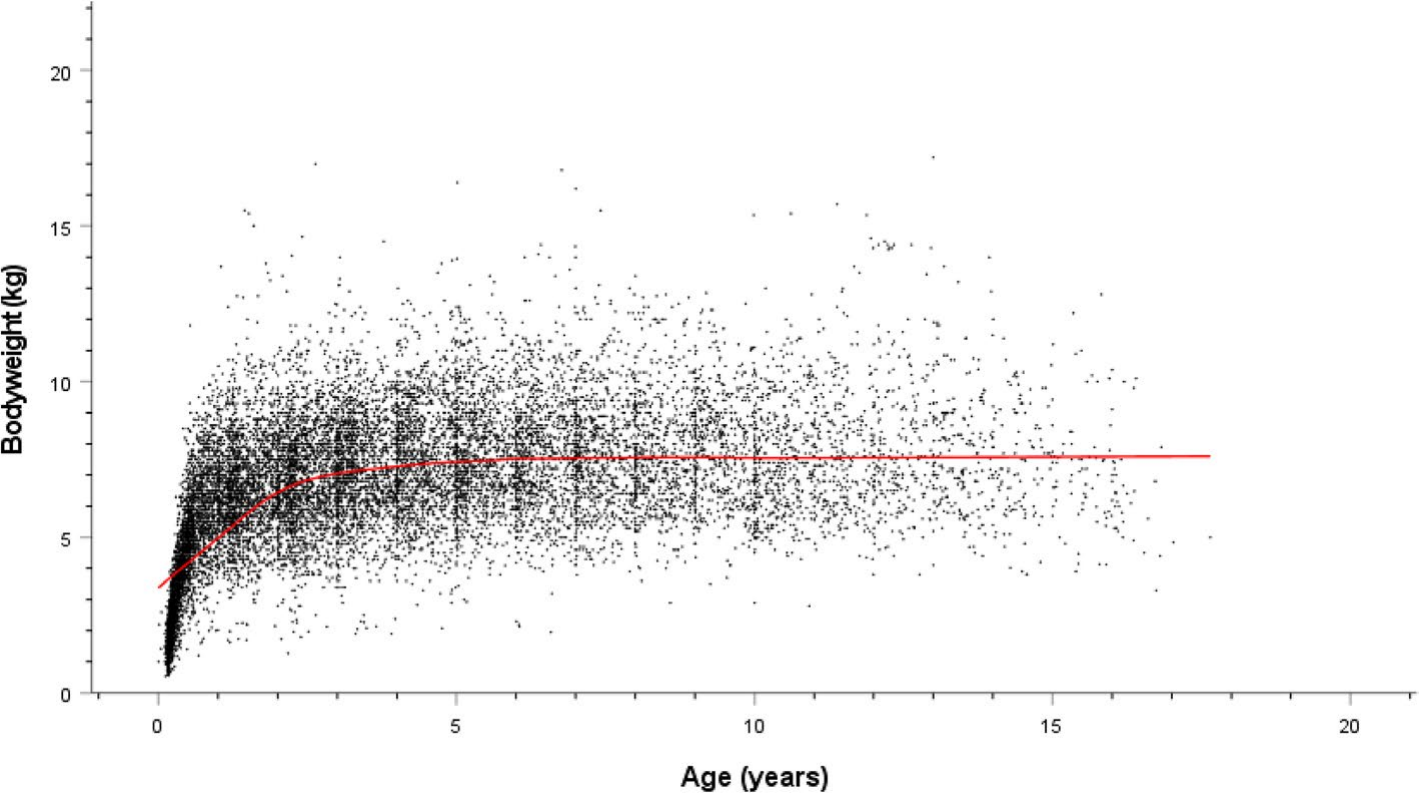
Bodyweight growth curve line plot overlaid with a loess line (uniform kernel, fitting 50% of points) for female Shih Tzus attending UK primary-care veterinary clinics participating in the VetCompass™ Programme

The study population of 336,865 dogs in the VetCompass™ database under veterinary care during 2016 included 11,082 (3.3%) Shih Tzus. The study included data from three large veterinary groups with 510 veterinary clinics spread across all areas of the UK. Of the 11,033 (99.6%) Shih Tzus with information available 5,276 (47.8%) were female and 4,315 (39.0%) were neutered. Males were more likely to be neutered than females (40.4% versus 37.7% respectively, *P* = 0.004). The mean adult bodyweight overall was 7.9 kg (SD: 1.9 kg). The mean adult bodyweight of males (8.5kg, SD: 1.9 kg) was heavier than for females (7.3 kg, SD: 1.7 kg) (*P* < 0.001) (Table 1). The median age of Shih Tzus overall was 4.1 years (IQR 2.1–7.1 range: 0.3–20.4). There was no significant difference in the median age of females (median 4.1 years; IQR 2.1–7.1; range 0.3–19.8) and males (median 4.0 years; IQR 2.1–7.2; range 0.4–20.4) (*P* = 0.498). Data completeness varied across the variables assessed: age 97%, sex 99.6%, neuter 100.0%, and adult bodyweight 67.3%. Annual proportional birth rates showed that Shih Tzus increased from 2.2% of the annual VetCompass birth cohort in 2005 to 3.8% in 2013 before dropping to 3.3% by 2016 (Fig. 1). Bodyweight growth curves based on 15,589 bodyweight values from 4,178 females and 18,429 bodyweight values from 4,772 males showed that Shih Tzus grow rapidly as puppies during their first year but continue to gain further bodyweight up to three years of age and that males weighed more than females (Figs. 2 and 3).

**Table 1:** Demography of Shih Tzus under primary veterinary care at practices participating in the VetCompass™ Programme in the UK from January 1^st^, 2016 to December 31^st^, 2016 (*n* = 11,082)

Variable	Category	Count^a^	Percent
Sex	Female	5,276	47.8
	Male	5,757	52.2
Female neuter	Entire	3,298	62.8
	Neutered	1,951	37.2
Male neuter	Entire	3,451	60.3
	Neutered	2,274	39.7
Female adult bodyweight (aged ≥ 18 months) (kg)	< 5.0	246	7.2
	5.0 to < 6.0	493	14.3
	6.0 to < 7.0	826	23.9
	7.0 to < 8.0	767	22.2
	8.0 to < 9.0	597	17.3
	9.0 to < 10.0	319	9.2
	≥ 10.0	203	5.9
Male adult bodyweight (aged ≥ 18 months) (kg)	< 5.0	76	1.9
	5.0 to < 6.0	212	5.4
	6.0 to < 7.0	527	13.3
	7.0 to < 8.0	822	20.7
	8.0 to < 9.0	838	21.2
	9.0 to < 10.0	673	17.0
	≥ 10.0	814	20.5
Age (years)	< 1.0	340	3.2
	1.0 to < 2.0	2,219	20.7
	2.0 to < 4.0	2,738	25.6
	4.0 to < 6.0	1,933	18.1
	6.0 to < 9.0	1,915	17.8
	≥ 9.0	1,123	14.6

^a^Count covers dogs with available data

### Disorder prevalence

The EPRs of a random subset of 2,423 Shih Tzus (21.9% of Shih Tzus) were manually examined to extract all recorded disorder data for 2016. There were 1,669 (68.9%) Shih Tzus with at least one disorder recorded during 2016 while the remaining 31.1% had no disorder recorded and either presented for prophylactic management only or did not present at all during 2016. The median annual disorder count per Shih Tzu during 2016 was 1 disorder (IQR 0–2, range 0–18). There was no significant difference in annual disorder count between males (median 1, IQR 0–2, range 0–14) and females (median 1, IQR 0–2, range 0–18) (*P* = 0.988).

The study included 3,779 unique disorder events recorded during 2016 encompassing 336 distinct fine-level disorder terms. The most prevalent fine-level precision disorders recorded in Shih Tzus were periodontal disease (*n* = 229, prevalence 9.5%, 95% CI: 8.4–10.7), anal sac impaction (180, 7.4%, 95% CI: 6.5–8.5) and ear disorder (134, 5.5%, 95% CI: 4.7–6.5). The median age of dogs diagnosed with individual fine-level disorders varied from 1.4 years (umbilical hernia) to 11.4 years (heart murmur). Males were more likely than females to be diagnosed with 4 of the 40 most common fine-level precision disorders (aggression, heart murmur, skin lesions and haircoat disorder) while females had higher prevalence than males for umbilical hernia (Table 2).

**Table 2:** Prevalence of the most common disorders at a fine-level of diagnostic precision recorded in Shih Tzus (*n* = 2,423) attending UK primary-care veterinary practices participating in the VetCompass™ Programme from January 1^st^, 2016 to December 31^st^, 2016

Fine-level disorder	Count	Overall prevalence %	95% CI^a^	Female prevalence %	Male prevalence %	*P*-Value	Median age (years)
Periodontal disease	229	9.5	8.4–10.7	10.5	8.4	0.080	6.8
Anal sac impaction	180	7.4	6.5–8.5	7.9	7.0	0.399	4.9
Ear disorder	134	5.5	4.7–6.5	4.7	6.4	0.058	4.2
Otitis externa	114	4.7	3.9–5.6	5.5	3.9	0.059	4.7
Vomiting	107	4.4	3.7–5.3	4.3	4.5	0.807	3.5
Overgrown nail(s)	103	4.3	3.5–5.1	3.8	4.6	0.337	3.7
Umbilical hernia	94	3.9	3.2–4.7	4.8	3.0	**0.018**	1.4
Post-operative complication	91	3.8	3.1–4.6	3.3	4.2	0.260	1.9
Obesity	86	3.5	2.9–4.4	3.5	3.6	0.863	4.4
Ulcerative keratitis (Corneal ulceration)	85	3.5	2.8–4.3	3.4	3.6	0.776	5.6
Diarrhoea	71	2.9	2.3–3.7	3.6	2.3	0.064	3.6
Flea infestation	62	2.6	2.0–3.3	2.8	2.2	0.344	3.6
Retained deciduous tooth	62	2.6	2.0–3.3	2.2	2.9	0.322	1.6
Aggression	59	2.4	1.9–3.1	1.5	3.2	**0.005**	4.5
Pododermatitis	56	2.3	1.8–3.0	1.7	2.9	0.064	3.6
Pruritus	56	2.3	1.8–3.0	2.7	2.0	0.263	3.2
Atopic dermatitis	54	2.2	1.7–2.9	2.2	2.2	0.971	4.9
Undesirable behaviour	53	2.2	1.7–2.9	2.3	2.1	0.650	3.5
Skin disorder	53	2.2	1.7–2.9	2.2	2.2	0.918	6.4
Ocular discharge	51	2.1	1.6–2.8	1.8	2.4	0.340	4.4
Heart murmur	51	2.1	1.6–2.8	1.5	2.7	**0.037**	11.4
Skin lesion(s)	51	2.1	1.6–2.8	1.5	2.7	**0.037**	3.9
Conjunctivitis	49	2.0	1.5–2.7	1.7	2.4	0.206	5.4
Haircoat disorder	49	2.0	1.5–2.7	1.2	2.8	**0.007**	4.2
Dental disease	46	1.9	1.4–2.5	1.4	2.4	0.080	7.7
Anorexia	45	1.9	1.4–2.5	2.3	1.4	0.091	4.1
Corneal disorder	45	1.9	1.4–2.5	1.6	2.1	0.308	6.4
Skin mass	42	1.7	1.3–2.3	1.4	2.1	0.225	5.5
Pyotraumatic dermatitis	38	1.6	1.2–2.2	1.2	2.0	0.109	2.5
Pyoderma	38	1.6	1.2–2.2	1.2	1.8	0.259	4.9
Skin cyst	38	1.6	1.2–2.2	1.7	1.5	0.720	6.4
Lameness	37	1.5	1.1–2.1	1.2	1.8	0.259	5.9
Mandibular prognathism	33	1.4	1.0–1.9	1.7	1.1	0.209	2.1
Anal sac infection	31	1.3	0.9–1.8	1.3	1.2	0.835	5.9
Keratoconjunctivitis sicca (KCS)	30	1.2	0.9–1.8	1.3	1.2	0.693	9.4
Wound	30	1.2	0.9–1.8	1.2	1.2	0.978	4.5
Gastroenteritis	29	1.2	0.8–1.7	1.1	1.3	0.594	3.2
Ophthalmic disorder	27	1.1	0.8–1.6	1.2	1.0	0.544	7.2
Claw injury	26	1.1	0.7–1.6	0.9	1.2	0.445	5.0
Thin	21	0.9	0.6–1.3	0.7	1.0	0.526	3.1

The *P*-value reflects prevalence comparison between females and males. Significant results in bold

^a^*CI* confidence interval

There were 53 distinct grouped-level precision disorder terms recorded. The most prevalent grouped-level precision disorders were cutaneous (*n* = 402, prevalence: 16.6%, 95% CI: 15.2–18.1), dental disorder (322, 13.3%, 95% CI: 12.0–14.7), ophthalmological disorder (289, 11.9%, 95% CI: 10.7–13.3), enteropathy (255, 10.5%, 95% CI: 9.4–11.8) and ear (aural) disorder (247, 10.2%, 95% CI: 9.1–11.5). The median age of dogs diagnosed with individual grouped-level disorders varied from 1.6 years (hernia) to 11.5 years (heart disease). Males were more likely than females to be diagnosed with 2 of the 20 most common grouped-level precision disorders (skin (cutaneous) disorder and musculoskeletal disorder) while females had higher prevalence than males for hernia (Table 3).

**Table 3:** Prevalence of the most common grouped-level disorders recorded in Shih Tzus (*n* = 2,423) attending UK primary-care veterinary practices participating in the VetCompass™ Programme from January 1^st^, 2016 to December 31^st^, 2016

Grouped-level disorder	Count	Overall prevalence %	95% CI^a^	Female prevalence %	Male prevalence %	*P*-Value	Median age
Skin disorder	402	16.6	15.2–18.1	14.6	18.6	**0.007**	4.3
Dental disorder	322	13.3	12.0–14.7	13.6	12.9	0.606	5.8
Ophthalmological disorder	289	11.9	10.7–13.3	11.6	12.3	0.635	6.2
Enteropathy	255	10.5	9.4–11.8	10.7	10.4	0.778	3.4
Ear disorder	247	10.2	9.1–11.5	10.1	10.2	0.958	4.5
Anal sac disorder	211	8.7	7.7–9.9	9.2	8.2	0.385	5.1
Behavioural disorder	153	6.3	5.4–7.4	5.7	6.8	0.231	3.6
Mass lesion	141	5.8	5.0–6.8	5.9	5.8	0.883	7.2
Claw disorder	137	5.7	4.8–6.6	4.8	6.4	0.088	4.1
Musculoskeletal disorder	118	4.9	4.1–5.8	3.7	6.0	**0.010**	6.2
Hernia	108	4.5	3.7–5.4	5.7	3.3	**0.005**	1.6
Complication associated with clinical care procedure	105	4.3	3.6–5.2	4.2	4.5	0.655	2.1
Parasite infestation	97	4.0	3.3–4.9	4.4	3.5	0.275	3.3
Upper respiratory tract disorder	87	3.6	2.9–4.4	3.7	3.5	0.708	2.4
Obesity	86	3.5	2.9–4.4	3.5	3.6	0.863	4.4
Heart disease	82	3.4	2.7–4.2	3.0	3.8	0.281	11.5
Neoplasia	65	2.7	2.1–3.4	3.1	2.3	0.241	7.9
Female reproductive abnormality (females only)	55	2.3	1.7–2.9	~	~	~	3.4
Traumatic injury	55	2.3	1.7–2.9	2.2	2.3	0.921	3.6
Appetite disorder	47	1.9	1.5–2.6	2.4	1.5	0.098	4.1

The *P*-value reflects prevalence comparison between females and males. Significant results in bold

^a^*CI* confidence interval

After combining the 10 most common disorders recorded at a fine-level of diagnostic precision within at least one of three age bands (under 2 years, 2–7 years and over 7 years), there were prevalence values reported for 16 disorders. Of the Shih Tzus with available age data (*n* = 2,365), there were 543 dogs aged under 2 years (22.96%), 1,227 dogs aged from 2 to 7 years (51.88%) and 595 dogs aged over 7 years (25.16%). The prevalence of nine of these 16 disorders (56.25%) differed statistically between the three age groups (Table 4).

**Table 4:** Prevalence of the combined list from the 10 most common disorders recorded at a fine-level of diagnostic precision within each of three age bands (under 2 years, 2–7 years, over 7 years) in Shih Tzus under primary veterinary care at UK practices participating in the VetCompass™ Programme from January 1st to December 31st, 2016

Fine-level disorder	< 2 yrs: Rank	< 2 yrs: N (%)	2–7 yrs: Rank	2–7 yrs: N (%)	> 7 yrs: Rank	> 7 yrs: N (%)	*P*-Value
Anal sac impaction	6	27 (4.97)	2	101 (8.23)	2	51 (8.57)	**0.032**
Dental disease	43	4 (0.74)	31	17 (1.39)	8	25 (4.20)	** < 0.001**
Diarrhoea	9	18 (3.31)	8	38 (3.10)	21	15 (2.52)	0.707
Ear disorder	4	31 (5.71)	4	63 (5.13)	4	39 (6.55)	0.465
Heart murmur	217	0 (0.00)	47	8 (0.65)	3	42 (7.06)	** < 0.001**
Umbilical hernia	1	58 (10.68)	21	22 (1.79)	36	8 (1.34)	** < 0.001**
Keratoconjunctivitis sicca (KCS)	123	1 (0.18)	51	7 (0.57)	10	22 (3.70)	** < 0.001**
Obesity	15	13 (2.39)	6	55 (4.48)	16	18 (3.03)	0.063
Otitis externa	10	18 (3.31)	3	66 (5.38)	6	29 (4.87)	0.170
Overgrown nail(s)	7	21 (3.87)	5	56 (4.56)	9	23 (3.87)	0.701
Flea infestation	8	20 (3.68)	13	33 (2.69)	34	9 (1.51)	0.071
Periodontal disease	26	8 (1.47)	1	111 (9.05)	1	109 (18.32)	** < 0.001**
Post-operative complication	3	45 (8.29)	9	36 (2.93)	35	9 (1.51)	** < 0.001**
Retained deciduous tooth	2	46 (8.47)	35	16 (1.30)	315	0 (0.00)	** < 0.001**
Ulcerative keratitis (Corneal ulceration)	22	9 (1.66)	10	36 (2.93)	5	35 (5.88)	** < 0.001**
Vomiting	5	31 (5.71)	7	47 (3.83)	7	27 (4.54)	0.207

The *P*-value reflects prevalence comparison between the three age bands using chi-squared tests. Under 2 years : *n* = 543, 2–7 years: *n* = 1,227, over 7 years: *n* = 595. Significant results in bold

### Mortality

There were 89 deaths recorded during the study of which 85 (95.5%) had a recorded age at death. Median age of death was 12.7 years (IQR 8.7–14.3, range 2.0–19.9). The median age at death of females (12.6 years, IQR 8.5–14.8, range 2.0–17.7, *n* = 48) did not differ significantly to that of males (12.7 years, IQR 10.0–14.2, range 2.0–19.9, *n* = 37) (Mann–Whitney U test: *P* = 0.697). Of 85 deaths (95.5%) where method of death was recorded, 80 (94.1%) deaths involved euthanasia and there were 5 (5.9%) unassisted deaths. Sixteen (18.0%) deaths did not have a biomedical cause of death stated. Of the remaining 73 deaths (82.0%), the most common causes of death described at a grouped-precision level were enteropathy (*n* = 7, prevalence 9.6%), heart (cardiac) disease (*n* = 7, prevalence 9.6%), and poor quality of life (*n* = 7, prevalence 9.6%) (Table 5).

**Table 5:** Mortality in Shih Tzus with a recorded cause of death under primary-care veterinary at UK practices participating in the VetCompass™ Programme from January 1^st^, 2013 to December 31^st^, 2016 (*n* = 89)

Grouped-level disorder	Count	Percent	95% CI
Enteropathy	7	7.9	3.9–15.4
Heart (cardiac) disease	7	7.9	3.9–15.4
Poor quality of life	7	7.9	3.9–15.4
Spinal cord finding	5	5.6	2.4–12.5
Appetite finding	5	5.6	2.4–12.5
Neoplasia	5	5.6	2.4–12.5
Thin finding	5	5.6	2.4–12.5
Behaviour disorder	4	4.5	1.8–11.0
Lower respiratory tract disorder finding	4	4.5	1.8–11.0
Collapsed	3	3.4	1.2–9.5
Ophthalmological disorder finding	3	3.4	1.2–9.5
Renal disease	3	3.4	1.2–9.5
Abdominal finding	2	2.2	0.6–7.8
Lethargy	2	2.2	0.6–7.8
Skin (cutaneous) disorder	2	2.2	0.6–7.8
Brain disorder	1	1.1	0.2–6.1
Complication associated with clinical care procedure finding	1	1.1	0.2–6.1
Hepatopathy (liver disorder)	1	1.1	0.2–6.1
Mass lesion finding	1	1.1	0.2–6.1
Musculoskeletal disorder	1	1.1	0.2–6.1
Neurological (nervous system) disorder (unspecified)	1	1.1	0.2–6.1
Syncope	1	1.1	0.2–6.1
Traumatic injury	1	1.1	0.2–6.1
Upper respiratory tract finding	1	1.1	0.2–6.1

## Discussion

Using primary-care veterinary data in the UK, this study described the demography of 11,082 Shih Tzus, and the disorder prevalence and mortality from 2,423 Shih Tzus. The annual proportional birth rates of Shih Tzus from 2005–2016 showed increasing popularity from 2005 to 2013 before dropping in 2016. The most prevalent fine-level disorders of Shih Tzus were periodontal disease, anal sac impaction and ear disorders. The most commonly reported grouped causes of mortality were enteropathy, heart disease and poor quality of life. The results demonstrate how primary-care veterinary records can be used to identify common health issues within dog breeds and can provide evidence to help clinicians prioritise breed-focused health and welfare considerations.

The current study identified rising annual proportional birth rates in Shih Tzus which nearly doubled from 2005 to 2013 (2.2%-3.8%) but showed signs of decreasing from 2013 to 2016. Shih Tzu registrations with the UK Kennel Club dropped by more than 50% from 4,319 in 2013 to just 1,906 in 2022 [3, 4]. The drop in popularity in Shih Tzus coincides with the rise in popularity of other brachycephalic breeds such as the French Bulldog which experienced a six-fold increase in Kennel Club registrations from 2013 (6,990) to 2022 (42,538) [3]. Prospective puppy owners who are drawn to the appeal of brachycephaly may be more likely to buy a French Bulldog than other brachycephalic breeds (such as Shih Tzus) due to promotion of French Bulldogs in marketing, social media, film and television [36]. While Shih Tzu popularity appears to be declining, the increased popularity of brachycephalic breeds in recent years is particularly concerning due to the strong evidence that brachycephalic dog breeds are generally less healthy than non-brachycephalic breeds [37]. Consequently, the UK Brachycephalic Working Group promotes the message ‘Stop and think before buying a flat-faced dog’ to encourage prospective owners to consider the health and welfare of dogs and to reduce the demand for dogs with brachycephalic conformation [38].

Periodontal disease was the most prevalent fine-level disorder of Shih Tzus, recorded in 9.5% of the population. This is comparable to the prevalence reported in breeds such as Labrador Retrievers (7.11%) [39], but lower than the prevalence reported in other dog breeds (e.g. West Highland White Terriers (15.7%), Border Terriers (17.63%), Greyhounds (39.0%)) [40–42]. These data are based on the same data source making the results more likely to be comparable. The prevalence of periodontal disease in Shih Tzus is lower than the reported prevalence of 12.5% in dogs overall attending primary-care practices in England in 2016 in a study with similar methodology to the present study [43], suggesting Shih Tzus are not predisposed to periodontal disease. Indeed, while a recent paper reported a one-year prevalence of periodontal disease of 12.52% in dogs in the UK, with brachycephalic breeds, including Shih Tzus, having 1.25 times the odds of periodontal disease compared mesocephalic breeds; Shih Tzus were not amongst the breeds found to be predisposed to periodontal disease in that study [44]. The current study also identified that the prevalence of periodontal disease significantly increased with age, with the highest prevalence in dogs aged over 7 years. Multiple studies across different dogs breeds have reported increasing odds of periodontal disease with age [45] and indeed that older dogs progress to periodontal disease faster than younger dogs [46], stressing the importance of oral hygiene in all ages but especially in dogs of advancing age. In the current study retained deciduous teeth were more prevalent in Shih Tzus under two years of age (8.47%) compared to Shih Tzus over two years (0.00–1.37%). This agrees with previous radiological examinations of dogs by Butković and colleagues (2001) who identified that retained deciduous teeth were most common in the youngest age group of dogs (seven months to two years of age) [47]. This may be logical because retained deciduous teeth are those which are not shed before the eruption of permanent teeth [48]. If not removed, retained primary teeth increase the likelihood of later dental disorders by contributing to the buildup of plaque which can predispose dogs to periodontal disease [49–51]. As periodontal disease was the most prevalent disorder in Shih Tzus in the current study, retained deciduous teeth were most common in young Shih Tzus, and retained deciduous teeth were the 13^th^ most common fine-level disorder in Shih Tzus, it stresses the importance of lifelong dental management in Shih Tzus and the need for careful planning and intervention [52].

In the current paper, anal sac impaction was the second most prevalent fine-level disorder, with 7.4% of Shih Tzus affected. This is similar, if slightly higher, than the prevalence reported in other breeds such as Chihuahuas (4.9%) and English Bulldogs (4.3%) [24, 53]. The prevalence of anal sac impaction in Shih Tzus in the current study (7.4%) was higher than the prevalence previously reported in dogs in the UK overall (4.8%) in a study with comparable methods [43], suggesting that Shih Tzus may have a predisposition for anal sac impaction. The prevalence of anal sac impaction diagnosis increased with increasing age, agreeing with previous research where anal sac disorders in UK dogs were found to significantly increase with advancing age over three years of age [11]. Anal sac impaction is a common disorder in dogs; the disorder was the fifth most commonly diagnosed fine-level disorder in dogs in a recently published cohort study into the one-year prevalence of disorders in dogs in the UK in 2016 [43]. The condition can be uncomfortable for dogs, leading to activities such as scooting or self-mutilation [54] to ease discomfort. Increased prevalence of anal sac disorders in Shih Tzus in particular has been previously reported in a study into non-neoplastic anal sac disorders of dogs in the UK; Shih Tzus were amongst the breeds with the highest prevalence of anal sac disorders (6.93%) and had 1.66 times the odds of being diagnosed with an anal sac disorder compared to crossbreed dogs suggesting a potential breed predisposition [11]. That study also identified a predisposition for brachycephalic breeds overall when conformation categories replaced breed in analyses [11]. Indeed, anal sac impaction has been identified as one of the most common disorders in brachycephalic breeds overall, and while the reasons for this are not entirely clear, the findings in the present paper adds to the literature suggesting reduced health in brachycephalic breeds compared to non-divergent dog breeds [37]. It has been suggested that diarrhoea may be a risk factor for anal sac impaction [55, 56] as dogs may be unable to naturally express their own anal sacs whilst defecating leading to build up and impaction [55]. In the present paper, enteropathy was among the most common disorders in Shih Tzus; it is possible that the high prevalence of enteropathy and diarrhoea in Shih Tzus could increase the likelihood of anal sac impaction. The findings of the current paper suggest the need for anal sac disorders to be viewed as a health priority for Shih Tzus, especially as the prevalence increases with increasing age.

The pattern of the most common disorders in Shih Tzus were similar to those previously reported in dogs overall in a recent study of dogs under primary veterinary care in the UK in 2016 [43] potentially suggesting that Shih Tzus can be considered as a relatively typical dog in terms of common health issues. However, while the common disorders of Shih Tzus were similar to the top disorders in dogs overall, the prevalence of these disorders in Shih Tzus differed from those previously reported in dogs overall in the UK [43]. For example, in addition to anal sac impaction, the prevalence of umbilical hernias in Shih Tzus in the current study was substantially higher (3.9%) compared to dogs overall (0.93%) [43], and similarly the prevalence of both ocular discharge and corneal ulceration were substantially higher (2.1% and 3.5%, respectively) in Shih Tzus in the current study than dogs overall (0.74% and 0.77%, respectively) [43]. With these additional comparisons, it is possible to suggest that Shih Tzus may have potential predispositions for anal sac impaction, umbilical hernias and eyes problems such as corneal ulceration and ocular discharge, adding to previous literature on Shih Tzus and dog breeds with brachycephaly [7, 11, 20]. Based on this previous evidence and the findings of the current paper, it may be worth considering whether the Breed Watch category status of Shih Tzus should be raised. As mentioned previously, Shih Tzus are currently classed as Breed Watch category 1, meaning the breed is considered to have no known health concerns which require special attention from judges [12]. However, as the current study has identified several potential predispositions, including eye problems related to their conformation [15, 16], it is possible that Shih Tzus could be moved to Breed Category 2 or 3, defined as breeds which have visual points of concern that may cause pain or discomfort to the dog, or breeds which can be considered to be susceptible to health problems associated with extreme conformations, respectively [57]. Moving Shih Tzus to a higher Breed Watch category may allow the health and welfare of Shih Tzus to be considered during judging and breeding more widely. It is important to consider that breed predispositions do not necessarily mean the disorder must also be considered a high priority for the breed in question; a predisposition may be low priority for a breed due to low prevalence or severity [58]. Awareness of breed predispositions can potentially help mitigation processes to be put in place to minimize negative welfare impacts.

The most common disorders of Shih Tzus (periodontal disease, anal sac impaction and ear disorders) are similar to those reported in dogs overall [43], but Shih Tzus appear to differ in their disorder profile compared to other brachycephalic breeds. A study which compared the disorders of brachycephalic breeds to non-brachycephalic breeds reported that the top disorders for brachycephalic breeds overall were periodontal disease, otitis externa, anal sac impaction, overgrown nails and diarrhoea [37]. While these are similar to the most common disorders reported in the current study, Shih Tzus had considerably lower prevalence of otitis externa (4.7% vs 7.27%) and obesity (3.5% vs 6.38%) [37]. Indeed, further differences can be observed when comparing Shih Tzus to other brachycephalic dog breeds individually. For example, the top disorders for French Bulldogs have been previously reported as otitis externa, diarrhoea and conjunctivitis according to a study with similar methodology to the current study [59]. While otitis externa was the fourth most common disorder in Shih Tzus in the current study, the prevalence in French Bulldogs (14%) [59] was more than double the prevalence observed in Shih Tzus (4.7%). Similarly, the prevalence of diarrhoea in French Bulldogs [59] was more than double the level observed in Shih Tzus in the current study, while conjunctivitis prevalences were similar. Shih Tzus also differ in their disorder profile from other brachycephalic breeds such as the Pug where top disorders include obesity, corneal disorders and otitis externa [60] at prevalences higher and in some cases more than double the prevalence observed in Shih Tzus. Bulldogs also differ from Shih Tzus with top disorders of otitis externa, pyoderma and obesity [61], again at prevalences more than double the prevalence of Shih Tzus. When looking at age of death, Shih Tzus appear to relatively long-lived compared to other brachycephalic breeds. The median age of death of Shih Tzus in the current study was 12.7 years which is higher than that reported in French Bulldogs (3.7 years) [59] and Bulldogs (7.2 years) [61], although some of the data from these studies are from young populations of dogs which may explain lower longevity estimates [59]. Based on this evidence, it is possible to suggest that the Shih Tzu can be considered not just as a brachycephalic breed but a breed with its own unique disorder profile which differs from other brachycephalic breeds with lower prevalence for many disorders commonly observed in these types of dogs.

There were some prevalence differences in Shih Tzus associated with sex and age. Female Shih Tzus were more likely to be diagnosed with an umbilical hernia than male Shih Tzus. Umbilical hernias are relatively uncommon in dogs overall; a study of dogs attending primary practice in the UK reported a prevalence of 0.93%, with umbilical hernia being the 33^rd^ most common disorder in dogs under primary-care in the UK in 2016 [43]. Shih Tzus have been reported to have a high within-breed prevalence of umbilical hernias [7]; however there has been no previous suggestion that these are more prevalent in female Shih Tzus [43]. The current study also observed a significant difference in umbilical hernia with age with a higher prevalence in young dogs aged under two years compared to older dogs. Hyun (2004) observed that hernias were more common in female and young dogs when radiographing cases but a conclusion could not be reached due to the small number of cases observed (42 dogs) [62]. The reason for this sex-related difference is not clear and warrants further investigation.

Male Shih Tzus in the current study were more likely to be diagnosed with heart murmurs than females, in agreement with male predispositions for cardiovascular conditions reported in previous studies [63, 64]. For example, a retrospective cross-sectional study of EPRs of dogs attending primary-care veterinary practice in the UK reported that male dogs had higher odds than females and Shih Tzus had higher odds than crossbreeds of having a degenerative mitral valve disease and heart murmur diagnosis [65]. A retrospective study by Serfass and colleagues (2006) on mitral valve disease in six small breeds of dogs (Yorkshire Terrier, Bichon Maltese, Dachshund, Poodle, Lhasa Apso and Shih Tzu) reported that Shih Tzus were among the most affected breeds. Shih Tzus also had a higher prevalence and progression of left apical systolic murmurs in male dogs compared to female dogs and the prevalence of murmurs increased with increasing age [66]. The current study also reported increased prevalence of heart murmurs with increasing age in agreement with previous research [67, 68]. The reason for the sex difference is yet to be ascertained and further research is required to investigate the effect of sex on cardiac disorder severity and age of onset [65]. Heart disease was also the second most common cause of death in the current study. A study of mortality from heart disease in insurance data from Swedish dogs under 10 years of age found that Shih Tzus were among the breeds with low mortality (15%) from heart disease. However, that insurance study investigated mortality from heart disease only in dogs under 10 years of age [69], and the current study reported an increased prevalence of heart murmurs with increasing age, with no age restrictions.

The current study identified that aggression was more prevalent in male Shih Tzus than females, with more than double the prevalence (3.2% vs 1.5% respectively). Aggression has been identified as being more prevalent in male dogs across several breeds of dogs e.g., Chihuahuas, Rottweilers and German Shepherds [24, 70, 71]. It has been suggested that male dogs may be more aggressive due to androgens which can lead to dominant- and competition-related aggression [72]. However, aggression was the 14^th^ most common fine level disorder in the Shih Tzu in the current study with an overall prevalence of 2.4% which is comparable to the prevalence of 2.24% reported in dogs overall in the UK in a similar study of dogs under attending primary-care veterinary practices [43] and in brachycephalic dogs overall (2.06%) [37], suggesting Shih Tzus should not be considered a particularly aggressive breed; Shih Tzus are described as ‘friendly’ on the Kennel Club website [73]. However, reporting and awareness of differences in health and disorder prevalence between male and female Shih Tzus can help veterinarians highlight which disorders may require more attention and signal to owners what to look out for in their male or female dog specifically.

Both keratoconjunctivitis sicca and ulcerative keratitis were more common in dogs aged over seven years old than younger dogs in the present study. Keratoconjunctivitis sicca has been associated with the development of corneal ulcerative disease [74]. As discussed above, brachycephalic dogs are strongly predisposed to a range of ophthalmic problems due to their extreme facial conformation [15, 17]. Indeed, corneal ulcerative disease is common in Shih Tzus; a recent study of EPRs of dog attending primary-care veterinary practices in the UK reported the Shih Tzu, at 3.45%, as the breed with the third highest prevalence, while Shih Tzus showed 10 times the odds of corneal ulcerative disease compared to crossbreed dogs. That study also reported that dogs aged three to more than 12 years of age had more than double the odds of corneal ulcerative disease compared to dogs under the age of three years [20]. The results of the current study agree with these findings and can be used to encourage owners and veterinarians to work together to reduce the welfare impacts from corneal ulcerative disease in Shih Tzus, especially as the disease burden increases as dogs age; for example, encouraging regular eye check-ups with age, and raising owner awareness of clinical signs to be vigilant for.

The prevalence of 10/16 most common disorders differed significantly across the three age groups of Shih Tzus (under 2 years, 2–7 years, over 7 years). Several disorders increased in prevalence with age, such as anal sac impaction, dental disease, heart murmur, keratoconjunctivitis sicca, periodontal disease, and ulcerative keratitis. Other disorders such as umbilical hernia, post operative complications and retained deciduous teeth were most common in the youngest age group and decreased with age. These findings are comparable to previous research. For example, a study which used Japanese insurance data reported that disorders including cardiovascular, ophthalmologic, and dental disorders increased in prevalence as dogs aged [75]. An older study based on insurance data in Sweden reported similar results with cardiovascular and ocular conditions alongside other disorders such as gastrointestinal, urinary, endocrine and neoplastic disorders increasing in prevalence with age, while traumatic injuries was most common in younger dogs, decreasing in prevalence with age [76]. However, it is challenging to compare results across studies with different methodologies due to varying methods of disorder categorisation. Knowledge of disorder prevalence across the lifespan of dog breeds can help owners and veterinarians implement preventative measures and disease surveillance as appropriate.

The median age of death of the Shih Tzus in this study was 12.7 years with no evidence of sex differences in mortality. This longevity estimate is slightly lower than a previous report of 13.3 years, but higher than the median age at death of 12.0 years reported across all dog breeds [4], suggesting Shih Tzus are generally long-lived dogs. The life expectancy of Shih Tzus at age zero has been estimated at 11.05 years according to life tables created using data from dogs under primary veterinary practice [6]. The median age of Shih Tzus in the current study was 4.1 years which is comparable to median age reported in other breeds e.g. English Cocker Spaniel (4.57 years) [77], Boxer (5.81 years) [78], Labrador Retriever (5.23 years) [39]. In the present study, the most common causes of death were enteropathy, heart (cardiac) disease and poor quality of life which are comparable to the patterns for the most common causes of death reported in other similar studies of dogs. For example, a cross-sectional study of UK Kennel Club breeds identified the most common causes of death in owned dogs according to owner-reported questionnaires as cancer, old age and cardiac conditions. In Shih Tzus, 18.1% died of cardiac conditions [79], while in a study with similar methodology to the current study, gastrointestinal and cardiac disorders were among the 15 most frequent causes of death of dogs overall in England between January 2009 and December 2011 [5]. In a study of primary-care data from euthanised dogs of all breeds under veterinary care in the UK, dogs which had a poor quality of life had 16.28 times the odds of being euthanised compared to dogs with neoplasia, whereas the most commonly recorded unassisted method of death was heart disease [80]. Only 89 dogs died in the current study, of which 85 had an age of death and 73 had a cause of death recorded. This small number of deaths in the analysis does suggest that these mortality data reported should be interpreted with caution. However, identification of the main causes of death in this population of Shih Tzus can provide veterinarians with disorders to monitor in the breed and take proactive steps to tackle in Shih Tzus.

This study used clinical records data from dogs under primary veterinary care. The limitations of using primary-care clinical data for epidemiological analyses have been previously discussed [24, 43, 81]. Additionally, the current study only includes data from dogs registered with a primary practice, meaning the data does not include unregistered dogs. The disorder prevalence in the unregistered dog population in the UK may potentially differ from the registered dog population [24] meaning the current results may not necessarily be relevant to the wider unregistered population. One factor to consider when reviewing studies on disorder occurrence is the difference between prevalence and predisposition. Prevalence describes the proportion of a population affected by a disorder while predisposition describes the prevalence of a disorder in one group or population compared to another group or population [58]. High prevalence does not necessarily mean a disorder is a predisposition for a breed and vice versa. Furthermore, whilst investigating disorder prevalence using primary-care data can provide insights into the health and welfare needs of breeds, a fuller picture of the potential welfare risk associated with disorders also requires ascertainment of severity and duration [82, 83]; however such information was not extracted in the current study. It is important to consider data source when comparing the results of the present study with other similar work in the field of veterinary research. For example, the current study reported an overall prevalence of ophthalmological disorders of 11.9% in Shih Tzus which is more than half the estimate of 24.7% reported in Shih Tzus in a study based on Japanese insurance data [75]. Appreciating and considering the differences between data sources and periods of study, in this case primary care data and insurance claim data, can help clarify results. For instance, the free-text clinical notes in primary care data can be investigated for further information regarding conditions whereas studies based on insurance data are often limited to clinical information provided when the insurance claims were made [84]. Insurance claim data may also not include information on all potential conditions as insurance policies can have certain exclusions such as pre-existing conditions [85]. VetCompass comprises clinical notes from primary-care veterinarians that often record clinical signs that describe the conditions being treated rather than the formal biomedical diagnoses [43]. Consequently, the prevalence of the true underlying biomedical causes of these conditions may be underestimated in the current results (e.g. diarrhoea may be recorded rather than campylobacteriosis). These results may be considered more representative of the contextualised care that is provided to dogs under UK primary veterinary practice [86]. The Royal College of Veterinary Surgeons (RCVS) has a register of veterinary surgeons; however, there is no complete national register of veterinary clinics in the UK. The RCVS maintains a register of veterinary practice premises accredited by the RCVS Practice Standards Scheme. In 2020, 3,235 premises were included in this register but the number is likely to be higher [87]. Compared to this, the current study included 510 clinics and therefore represents less than 20% of all clinics in the UK. This may mean that there is potential selection bias for the clinic, veterinary professional and owner demographics included in the current analysis. Since the current study was conducted, VetCompass has continued to accrue more practices to participate in this national welfare-focused research programme and currently has over 1,800 UK participating clinics [29]. Consequently, newer VetCompass studies could mitigate any selection biases within the current work.

## Conclusion

This study reported the demography, disorder prevalence and mortality of Shih Tzus under primary veterinary care in the UK. The most common disorders of Shih Tzus included periodontal disease, anal sac impaction, ear disorders, and otitis externa, reflecting the literature on the most common health problems of dogs overall, implying Shih Tzus may be considered a reasonably typical breed in terms of health issues. However, this study identified that the prevalence of anal sac impaction, umbilical hernias and eyes problems were substantially higher than previously identified in dogs overall [37, 43] suggesting Shih Tzus may have predispositions for these disorders. The most common causes of death were enteropathy, heart disease and poor quality of life. Sex differences were identified, with males having higher prevalence of aggression, heart murmur, skin lesions, and haircoat disorders, while females had a higher prevalence of hernias. Furthermore, disorder prevalence differed depending on age for several disorders; for example the prevalence of dental disease, periodontal disease and dry eye increased with age stressing the importance of routine canine health monitoring and regular dental check-ups for Shih Tzus throughout their lives. The findings of the current study can be used by veterinarians as an evidence-base on Shih Tzu health and can help them to identify which disorders to pay special attention to when treating Shih Tzus. For example, veterinarians could increase their focus on dental care in Shih Tzus of advancing age, and management of potential predispositions. Furthermore, veterinarians can use the findings of the current study to encourage current and prospective owners to prioritise the welfare of their dogs and to be vigilant of the potential health issues of Shih Tzus identified here. Breeders of Shih Tzus can similarly use these findings to advise owners about potential health problems to monitor in their new puppies. Additionally, breeders can help to protect future generations of Shih Tzus from potential pain, suffering, injury or disease by breeding responsibly and ensuring they only breed from healthy animals; adhering to The Animal Welfare (Licensing of Activities Involving Animals) Regulations 2018 that states that ‘No dog may be kept for breeding if it can reasonably be expected, on the basis of its genotype, phenotype or state of health that breeding from it could have a detrimental effect on its health or welfare or that of its offspring’ [88]. In summary, the current paper has highlighted several disorders which should be monitored and managed in Shih Tzus to protect the welfare of both current and future dogs.

## Data Availability

The datasets generated during and/or analysed during the current study will be made available at the RVC Research Online repository.

## Abbreviations

CI: Confidence interval
EPR: Electronic patient record
IQR: Interquartile range
KC: The Kennel Club
OR: Odds ratio
UK: United Kingdom
US: United States
